# Genetic characterization of LEP and TG5 gene polymorphisms in crossbred beef cattle populations

**DOI:** 10.5455/javar.2024.k849

**Published:** 2024-12-27

**Authors:** Dinh Van Dung, Duong Thi Huong, Than Thi Thanh Tra, Le Thi Thu Hang, Le Dinh Phung, Nguyen Huu Van, Ho Le Quynh Chau

**Affiliations:** University of Agriculture and Forestry, Hue University, Hue City, Vietnam

**Keywords:** Cattle, polymorphism, leptin, thyroglobulin, PCR-RFLP

## Abstract

**Objective:**

This study aimed to investigate the single nucleotide polymorphisms (SNPs) in the intron 2 region of the leptin (LEP) gene and the 5’ untranslated region of the thyroglobulin (TG5) gene across four crossbred beef cattle populations, including Blanc Bleu Belge × Lai Brahman (BLB), Charolais × Lai Brahman (CLB), Droughtmaster × Lai Brahman (DLB), and Red Angus × Lai Brahman (RLB) raised in Central Vietnam.

**Materials and Methods:**

A total of 200 tail hair root samples (50 per group) were collected, and genomic DNA was extracted. The PCR-RFLP method was utilized to analyze the LEP and TG5 gene polymorphisms using the restriction enzymes *Sau*3AI and *Psu*I, respectively.

**Results:**

The SNPs of LEP/*Sau*3AI and TG5/*Psu*I were present in all populations, with a lower frequency of the LEPB allele compared to LEPA. The LEPAA genotype was most common, followed by LEPAB and LEPBB; notably, the LEPBB genotype was absent in the CLB group. The Hardy–Weinberg equilibrium was observed for LEP/*Sau*3AI in the CLB and BLB populations. Conversely, the TG5CT genotype dominated all groups, with no individuals exhibiting the TG5TT genotype. None of the populations achieved Hardy–Weinberg equilibrium for TG5/*Psu*I. The level of polymorphism was moderate for LEP/*Sau*3AI in RLB and BLB and for TG5/*Psu*I across all groups.

**Conclusion:**

The SNPs of LEP/*Sau*3AI and TG5/*Psu*I may serve as valuable tools for genomic selection. By focusing on increasing the frequency of the TG5T allele, breeding programs can more effectively enhance beef marbling and other important traits, leading to improved beef quality and greater economic outcomes in the cattle industry.

## Introduction

The prevalence of crossbred beef cattle has surged in Vietnam. To enhance beef productivity, the Lai Brahman breed (a hybrid of Brahman bulls and local cows) is commonly used as the maternal line in crosses with beef breeds such as Blanc Bleu Belge, Brahman, Droughtmaster, Red Angus, or Charolais. However, existing breeding programs primarily focus on performance and carcass yield, while improvements in beef quality remain limited.

Beef carcass and quality are influenced by genetic and environmental factors [1]. The association of single nucleotide polymorphisms (SNPs) in candidate genes and carcass traits, beef quality, has been established [2–5]. The LEP gene in cattle comprises 3 exons separated by 2 introns [6]. The SNP LEP/*Sau*3AI involves a substitution of cytosine (C) with thymine (T), leading to an amino acid change from arginine to cysteine at position 2059 of the protein [7]. This allelic variation has influenced fat and protein yield, as well as age at first calving [7]. The TG gene codes for thyroglobulin, which is a glycoprotein hormone produced by thyroid follicular cells. A cytosine-to-thymine (C to T) substitution at nucleotide position 442 in the 5’ untranslated region (5’ UTR) of the TG gene (TG5) has been associated with enhanced intramuscular fat in beef cattle [8]. Polymorphisms in the LEP and TG5 genes are used as molecular markers for selecting marbling traits in various beef cattle breeds [9,10].

However, the former result of Putra et al. [11] showed that LEP and TG5 gene polymorphisms did not appear in Pasundan cattle in West Java. Therefore, identifying the genetic characteristics of these two genes in each beef cattle population is essential.

Currently, there is limited direct research on the LEP and TG5 gene polymorphisms in cattle populations raised in Vietnam, particularly in crossbred populations from Central Vietnam. There is a lack of comprehensive data regarding the specific polymorphisms of these genes in Vietnamese cattle. Additionally, the potential of these genetic markers to improve beef quality and breeding programs in Vietnamese cattle populations remains underexplored. This gap hinders the ability to develop targeted genetic selection programs aimed at enhancing productivity and meat quality in the region’s cattle herds. The objective of this study was to investigate and characterize the polymorphisms of the LEP and TG5 genes in four crossbred beef cattle populations raised in Central Vietnam, including BLB, CLB, DLB, and RLB. The findings from this study are expected to provide valuable insights that can help develop marker-assisted selection programs, ultimately improving both beef productivity and quality to enhance cattle performance and meet the needs of the beef industry.

## Materials and Methods

### Ethical statement

The research protocol was agreed upon by the Hue University Scientific Committee (Decision No. 1472/QD-DHH). All possible measures were taken during the sample collection to minimize animal distress and reduce the number of samples used.

### Collection of samples and extraction of genomic DNA

Two hundred tail hair root samples were collected from 200 healthy females representing four crossbred cattle genotypes, including Blanc Bleu Belge × Lai Brahman (BLB), Charolais × Lai Brahman (CLB), Droughtmaster × Lai Brahman (DLB), and Red Angus × Lai Brahman (RLB). Samples were obtained from various households in Quang Ngai province, Vietnam (latitude 15.12047 North and longitude 108.79232 East), with 50 samples collected from each population. Each sample was kept in a separate polythene bag and transferred to the laboratory for processing.

Genomic DNA was extracted from tail hair roots using the AccuRive sDNA/RNA Prep kit (KT manufacturer, Vietnam). To improve extraction efficiency, tail hair roots were homogenized in lysis buffer for 5 min using a Bullet blender (Next Advance, USA). Subsequent steps were carried out according to the instructions of the kit manufacturer. DNA concentration and quality were evaluated using a NanoDrop system (Thermo Scientific, USA).

### Genetic polymorphism analysis

Two specific PCR assays were conducted to amplify the 422 bp (LEP gene) and 545 bp (TG5 gene) fragments using two primer pairs (Table 1) described in previous studies [8,12]. PCR reactions were conducted in a thermal cycler (Axygen^®^ MaxyGene™, USA). In a total reaction volume of 20 μl, the following components were included: 50 ng of template DNA, 200 mM of dNTP, 1.25 µM of primer, 0.75 units of Taq polymerase, and 1× PCR buffer (Solgent, Korea). The PCR program included an initial 5-min denaturation at 95°C, followed by 35 cycles of 95°C for 40 sec, 62°C (LEP) or 55°C (TG5) for 40 sec, 72°C for 40 sec, and a final extension at 72°C for 7 min (LEP) or 10 min (TG5). The PCR products were separated on a 2.0% agarose gel containing 6× GelRed (ABT, Vietnam) and visualized using a Gel Doc™ XR+ (Bio-Rad, USA).

For restriction fragment length polymorphism (RFLP) analysis, a 12-μl reaction mix was prepared for each sample, consisting of 10-μl PCR product, 3 units of restriction enzyme, and 1× buffer. The restriction endonuclease *Sau*3AI was used to cleave the sequence 5’-GATC-3’ in the LEP gene, and the *Psu*I was used to cut the sequence 5’-R^GATCY-3’ in the TG5 gene. The RFLP reactions were digested overnight at 37°C. Finally, the RFLP products were visualized using 2.0% agarose gels and captured in Gel DocTM XR+ (BioRad, USA) with 6× GelRed (ABT, Vietnam). The LEP/*Sau*3AI genotypes were classified as LEPAA (390 and 32 bp), LEPAB (390, 303, 88, and 32 bp), and LEPBB (303, 88, and 32 bp) [13]. Meanwhile, the TG5/*Psu*I genotypes were classified as TG5CC (295 bp, 178 bp, and 72 bp), TG5CT (473 bp, 295 bp, 178 bp, and 72 bp), and TG5TT (473 bp and 72 bp) [14].

**Table 1.** Primers used for amplification of LEP and TG5 genes.

Gene	SNP	Location	Primer sequence (5’-3’)	Amplicon (bp)	GenBank	References
LEP	g.1926C>T	Intron 2	LEPF: TGGAGTGGCTTGTTATTTTCTTCT	422	EU313203	[12]
			LEPR: GTCCCCGCTTCTGGCTACCTAACT			
TG5	g.422C>T	5’UTR	TG5F: GGGGATGACTACGAGTATGACTG	545	AY615525.1	[8]
			TG5R: GTGAAAATCTTGTGGAGGCTGTA			

### Data analysis

The genotype frequency, allele frequency, expected heterozygosity (H_e_), observed heterozygote (H_o_), and effective allele numbers (N_e_) were calculated.

The observed heterozygosity (H_o_) was determined as follows:

H_o_ = n/N

where n is the number of heterozygous animals for the allele and N is the sample size.

The expected heterozygosity (H_e_) was calculated using the formula:


$$H_{e}\;=\;1-\sum_{p_{i}}^{2}0$$

where p_i_ is the frequency of the ith allele.

The number of effective alleles (N_e_) was calculated using the formula:


Ne=1∑p2

where p_i_ is the frequency of the ith allele.

Polymorphic informative content (PIC) was calculated as follows

where p_i_ is the frequency of the ith allele at the marker locus and n is the number of different alleles. The chi-squared (χ^2^) test was used to evaluate whether the allele and genotype frequencies of both SNPs in each cattle group deviated from the Hardy–Weinberg equilibrium.

## Results

### LEP/Sau3AI polymorphism

**Figure 1.**
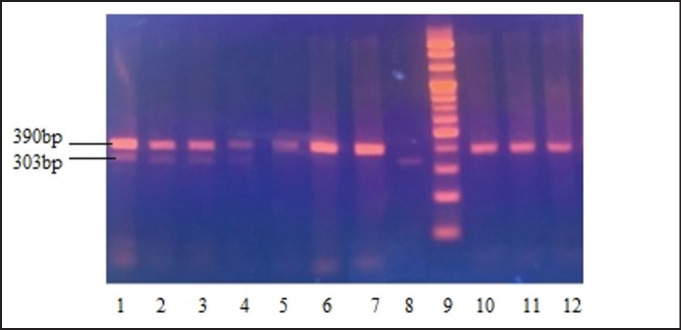
Result of PCR-RFLP analysis LEP/*Sau*3AI on 2% agarose gel. Lanes 1–4: LEPAB genotype; Lanes 5–7 and 10–12: LEPAA genotype; Lane 8: LEPBB genotype; Lane 9: 100 bp DNA ladder marker.

A 422-bp fragment of the LEP gene was successfully amplified from the genomic DNA of four crossbred cattle groups (Fig. 1). After digesting the PCR products with the *Sau*3AI enzyme, an SNP was detected, which resulted in the identification of three genotypes, including LEPAA, LEPAB, and LEPBB. The observed frequencies for the LEPA and LEPB alleles ranged from 0.53 to 0.89 and from 0.11 to 0.47, respectively (Table 2), indicating that the LEPA allele was more prevalent across all four populations. The LEPAA genotype was the most common in all groups, followed by LEPAB and LEPBB. Notably, the LEPBB genotype was absent in the CLB group, while genotype frequencies in the RLB group were similar.

The expected frequencies of the LEPAA, LEPAB, and LEPBB genotypes in the four cattle groups were calculated based on the Hardy–Weinberg formulas. The expected frequency of LEPAA was dominant in both the CBL and DLB groups, with LEPAB and LEPBB following. In contrast, the RLB group exhibited the highest expected frequency of LEPAB, with LEPAA and LEPBB genotypes being comparable. Overall, the LEPBB genotype showed the lowest expected frequency across all groups. The expected heterozygosity (He) was highest in the RLB group, while the observed heterozygosity (Ho) was greatest in the BLB group. The χ² test results (Table 2) indicated that the LEP/*Sau*3AI gene in BLB and CBL populations adhered to the Hardy–Weinberg equilibrium. The polymorphic information content (PIC), which reflects polymorphism levels, was categorized as low (PIC < 0.25) in CBL and DLB and moderate (0.25 < PIC < 0.50) in the BLB and RLB groups (Table 2). Consequently, the polymorphism of the LEP/*Sau*3AI gene is considered appropriate for molecular selection in BLB and RLB cattle populations.

**Table 2.** Polymorphism of LEP/*Sau*3AI gene in four crossbred populations (*n =* 50).

Crossbred population	Observed genotype frequency			Observed allele frequency		Expected genotype frequency			H_o_	H_e_	N_e_	PIC	HWE (χ^2^)
	AA	AB	BB	A	B	AA	AB	BB					
BLB	0.50	0.40	0.10	0.70	0.30	0.49	0.42	0.09	0.40	0.42	2.50	0.33	0.11^*^
CLB	0.76	0.24	0.00	0.88	0.12	0.77	0.21	0.01	0.24	0.21	4.17	0.19	0.93^*^
DLB	0.82	0.14	0.04	0.89	0.11	0.79	0.20	0.01	0.14	0.20	7.14	0.18	4.06
RLB	0.36	0.34	0.30	0.53	0.47	0.28	0.50	0.22	0.34	0.50	2.94	0.37	5.04

BLB: BBB × Lai Brahman; CLB: Charolais × Lai Brahman; DLB: Droughtmaster × Lai Brahman; RLB: Red Angus × Lai Brahman; Ho: observe heterozygosity; He: expected heterozygosity; Ne: number of effective alleles; PIC: polymorphic informative content; HWE: Hardy–Weinberg Equilibrium; χ^2^: chi-square value; *genetic equilibrium (*χ*^2^ < 3.84).

**Figure 2.**
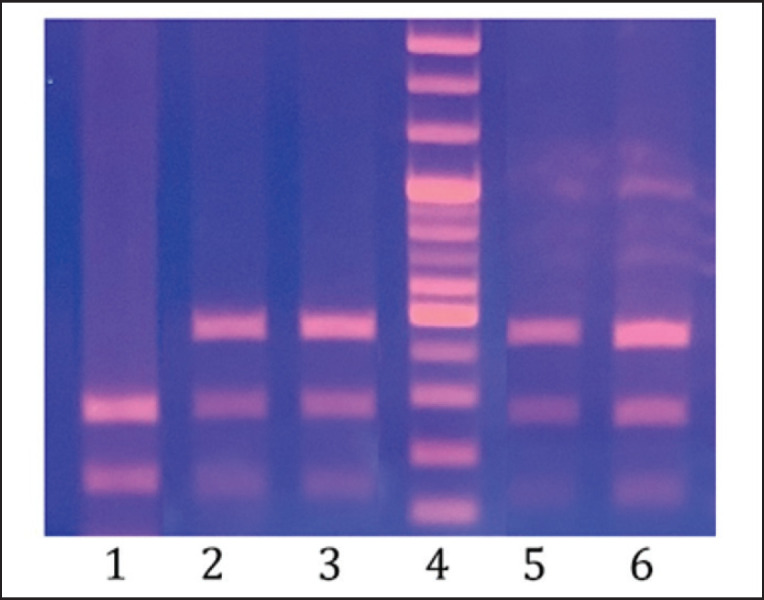
Result of PCR-RFLP analysis TG5/PsuI on 2% agarose gel. Lanes 1: TG5CC genotype; Lane 2,3,5 and 6: TG5CT genotype; Lane 4: 100 bp DNA ladder marker.

### TG5/PsuI polymorphism

The 545-bp fragment of the TG5 gene was successfully amplified, revealing the presence of polymorphism in the TG5/*Psu*I region across all four crossbred groups (Fig. 2). Most surveyed cattle were heterozygous for the TG5CT genotype, with none exhibiting the homozygous TG5TT genotype. Expected frequencies of the TG5TT genotype, based on Hardy–Weinberg calculations, ranged from 0.06 to 0.20 across groups. In all four populations, the observed heterozygosity coefficient exceeded the expected heterozygosity coefficient. None of the four cattle populations reached the Hardy–Weinberg equilibrium for the investigated locus (Table 3). The TG5/*Psu*I polymorphism exhibited a moderate PIC value and is suitable for molecular selection in these populations.

## Discussion

Previously published research results showed the difference in body weight among crossbred cattle. In household conditions, the BLB crossbred cattle have the highest body weight, followed by CBL, RLB, and DLB [15,16]. Consistent with our findings, previous studies have reported that the LEPA allele is more prevalent than the LEPB allele in various cattle populations [13,17]. Studies on the polymorphisms of the LEP gene in Hereford, Limousin, Iraqi, and Holstein Frisian cattle have also identified two primary genotypes of the LEP gene, including AA and AB [13,17]. The SNP in the LEP/*Sau*3AI gene has a notable effect on milk yield and age at first calving [6]. Cows with the LEPAA genotype demonstrate the highest milk yield, along with greater protein and fat content, and the earliest age at first calving. On the other hand, the LEPBB genotype is linked to the lowest milk production, while the LEPAB genotype is associated with the oldest age at first calving [6]. Additionally, Yang et al. [18] noted that cattle with the LEPBB genotype exhibited superior growth in Chinese indigenous breeds. Therefore, breeding strategies should prioritize the LEPAA genotype for enhancing reproductive traits and the LEPBB genotype for growth performance improvement.

Furthermore, Barendse et al. [19] identified TG5/*Psu*I as a key genetic variant associated with intramuscular fat content, where the TG5T allele was linked to improved marbling. Previous studies reported a low frequency of the TG5T allele in Brahman cattle (0.030). Multiple studies have also highlighted the impact of TG5/*Psu*I polymorphism on backfat thickness ribeye area [9] and intramuscular fat [20]. Furthermore, Barendse et al. [19] identified TG5/*Psu*I as a key genetic variant associated with intramuscular fat content, where the TG5T allele was linked to improved marbling. Previous research found that the TG5T allele was present at a rate of 0.030 in Brahman cattle [9] and lower frequencies in *Bos taurus indicus* (0.077–0.120) compared to *Bos taurus taurus* (0.138–0.286) [21]. Similarly, Rivera-Prieto et al. [8] identified a higher frequency of the TG5T allele (0.548) compared to the TG5C allele (0.452) in Beefmaster cattle, a hybrid of *Bos taurus* and *B. indicus*. Fortes et al. [22] observed an increasing presence of the TG5T allele with a higher *B. taurus* influence. In German Holstein and Charolais cattle, homozygous TG5TT individuals had considerably more fat in the loin muscle compared to those with the TG5CT and TG5CC carriers [19].

**Table 3.** Polymorphism of TG5/*Psu*I gene in four crossbred populations (*n =* 50).

Crossbred population	Observed genotype frequency			Observed allele frequency		Expected genotype frequency			H_o_	H_e_	N_e_	PIC	HWE (χ^2^)
	CC	CT	TT	C	T	CC	CT	TT					
BLB	0.16	0.84	0.00	0.58	0.42	0.34	0.49	0.17	0.84	0.49	1.19	0.37	26.22
CLB	0.50	0.50	0.00	0.75	0.25	0.56	0.38	0.06	0.50	0.38	2.00	0.30	5.56
DLB	0.18	0.82	0.00	0.59	0.41	0.35	0.48	0.17	0.82	0.48	1.22	0.37	24.15
RLB	0.10	0.90	0.00	0.55	0.45	0.30	0.50	0.20	0.90	0.50	1.11	0.37	33.47

BLB: BBB × Lai Brahman; CLB: Charolais × Lai Brahman; DLB: Droughtmaster × Lai Brahman; RLB: Red Angus × Lai Brahman; Ho: observe heterozygosity; He: expected heterozygosity; Ne: number of effective alleles; PIC: polymorphic informative content; HWE: Hardy–Weinberg Equilibrium; χ^2^: chi-square value.

Burrell et al. [23] also reported that TG5TT genotype cattle had superior marbling scores. The TG5T allele was more prevalent in cows with high marbling scores, and the TG5TT genotype was the only one associated with increased marbling in beef, showing distinct differences in marbling distribution [20]. Furthermore, Van Eenennaam et al. [24] confirmed an increased presence of the TG5T allele among higher graded carcasses. Recently, Sycheva et al. [25] indicated that the TG5 (g.422C > T) polymorphism significantly (*p* < 0.05) influenced the differentiation of Simmental bulls, affecting the linolenic acid, stearic acid, and total polyunsaturated fatty acid synthesis, as well as the dry matter and fat content in the *longissimus dorsi *muscle. Carriers of the TG5T allele showed an increase in polyunsaturated fatty acid synthesis, positioning the TG5T allele as a “desirable” allele [25]. The low frequencies of the TG5T allele in the SNP TG5/*Bst*YI (ranging from 0.0% to 1.5%) and the absence of the homozygous TG5TT genotype in seven Vietnamese native cattle populations were also reported [26]. The observed TG5T allele frequencies in this study, ranging from 0.25 to 0.45, suggest a moderate presence of this allele within the investigated cattle populations. This moderate frequency indicates that the TG5T allele is an integral component of the genetic diversity in these populations, and it could have significant implications for genetic improvement programs focused on beef quality.

The adherence of LEP/*Sau*3AI gene polymorphism to the Hardy–Weinberg equilibrium in the BLB and CLB populations suggests that these groups are in genetic equilibrium, meaning that the allele and genotype frequencies remain stable over generations. It implies that there are no significant influences from mutation, migration, or genetic drift affecting the LEP gene in these specific cattle populations, making them suitable candidates for genetic studies and selection programs aimed at improving traits like beef quality. Nevertheless, the failure of the TG5/*Psu*I gene polymorphism to achieve the Hardy–Weinberg equilibrium in all investigated populations suggests the presence of underlying factors influencing allele and genotype frequencies. This finding suggests that the TG5 gene may be under some form of evolutionary influence in these populations, which could impact future breeding strategies aimed at improving traits like marbling. Further investigation into mating patterns, selection practices, and population structure would be beneficial to understand the dynamics at play.

## Conclusion

Polymorphisms in the LEP/*Sau*3AI and TG5/*Psu*I genes were observed in all four investigated cattle populations. The LEPA allele had a higher frequency than the LEPB allele in all four cattle populations. The desired TG5T allele was present at moderate frequencies across the studied cattle populations. These SNPs can serve as candidate genes for marker-assisted selection to enhance reproductive performance and marbling quality in cattle, with LEP/*Sau*3AI being particularly suitable for RLB and BLB cattle and TG5/*Psu*I applicable across all investigated populations.

## List of abbreviations

BLB, BBB × Lai Brahman; bp, base pair; CLB, Charolais × Lai Brahman; DLB, Droughtmaster × Lai Brahman; He, expected heterozygosity; Ho, observe heterozygosity; HWE, Hardy–Weinberg Equilibrium; LEP, leptin; Ne, number of effective alleles; PIC, polymorphic informative content; Red Angus × Lai Brahman; RLB, Red Angus × Lai Brahman; SNP, single nucleotide polymorphism; TG5, 5’ untranslated region of thyroglobulin; χ^2^, chi-square value.
